# Patriotism and Nurse-Training Schools

**Published:** 1916-07-15

**Authors:** 


### PATRIOTISM AND NURSE-TRAINING SCHOOLS.

### VII. Training Schools South and West.

The proximity of the southern and western hospitals of the kingdom to the ports of landing has led to their extensive occupation by the wounded, and has largely retained the nursing staff in their own institutions. There are, however, exceptions, and in some of these institutions large numbers of nurses have gone to military hospitals at home or abroad, in spite of additions made to the wards which have increased the work.

### Royal Sussex County Hospital, Brighton.

Nearly eighty nurses holding the certificate of the Sussex County Training School are or have been doing military nursing. Out of the private staff alone there are at present thirty-two in military hospitals or hospital ships. Nearly all the sisters have been serving, and the matron has been able to provide many younger ones for temporary and permanent posts. Several of the nurses who had left came back to help through a difficult time. The following sisters and nurses went straight from the hospital staff for military service, and are now or have been in the localities named:—

*Salonika.*—Sister Davidge. *Malta.*—Sister Miller. *Mesopotamia.*—Sister Stagg.

*Alexandria.*—Sister Bates, Sister Earl, Sister Nelson; Nurses Clark and Dennehy. France.—Sister H. Panton; Nurses L. Clayton and Eager. *Serbia.*—Nurses A. Gambier, L. Hervey, R. Thomas, H. Willis. *Hospital Ship.*—Sister P. Roberts; Nurses M. Poole and Oppenheim. *Home Service.*—Sisters Nightingale, Paterson, M. Victor, Hesketh, Burton, Martin, and Staveley; Nurse Berger.

The following members of the private staff have left for the period of the war:—*France.*—Sister Leppard; Nurses Mills, Dixon, Throwgood, and Poulters. *Alexandria.*—Sister Brinnand; Nurses Bentham and Jameson. *Hospital Ship.*—Nurses Steele, Horsey, and McCulloch. *Serbia.*—Nurse Scamell. *Malta.*—Sister Duncan and Nurse Stroud. *Home Service.*—Sisters Biggs, Murray, Angus, and Storar; Nurses Dovet, Woodruff, Maddox, Duke, Cheal, Harrison, Stilwell, Gerrard, Taylor, Patterson, Ealand, Woods, Blaker, Childs, and Young.

Nurse Vivian Bury died of typhoid fever after a few months in Serbia. Four sisters holding this certificate have gained the Royal Red Cross:—*First Class*, Miss K. Mann; *Second Class*, Miss B. D. Turner, Miss E. R. Draper, Miss M. M. Staveley. Several nurses holding the R.S.C.H. Certificate are acting as matrons—viz., Mrs. Wisden (late Sister Shotter), Sister Foster, and Sister Scott Ram, Q.A.I.M.N.S.; Sister Mann and Sister Phillips are on hospital ships.

### Bristol General Hospital.

Twenty-three members of the staff are at present employed by the Navy and Army for service during the war. These comprise the following:—

*Naval.*—Nurse Howe, at the Royal Naval Hospital, Chatham; Nurse Lund, R.N. Hospital, Deal; Nurses Tobin, Kitley, and Newcombe, at the Royal Naval Hospital, Plymouth. *Military.*—Sister Sampson, in France; Sister Keene, matron of hospital ship; Sister Cox, in France; Nurses Wright, Baugh, Willcox, Hill, and Iles, nursing in France; Nurses Gough, Bodey, Carter, Evans-Harries, Poole, Williams, Smith, and Coomber, nursing in Egypt. Nurses Passmore and Gardiner, on hospital ships. Eleven nurses from the private nursing staff were engaged by the War Office in the autumn of 1915 and sent to Malta.

### Royal Devon and Exeter Hospital, Exeter.

Miss E. Smale, R.R.C., matron of the Royal Devon, is now principal matron of the 4th Southern General Territorial Hospital at Plymouth. Sisters Martin, Jones, Phillips, and Fell are working at that hospital with Nurses Sinclair and Lewis. In addition to this surrender made for the wounded by the hospital, the following other members of the staff are now doing military nursing: *Navy.*—Nurses Browne, Morgan, Richardson, Sawtell, and Cumberland. *Army.*—Nurses Swain, Locke, Bassett, Paterson, M. Gregory, Coates, Bursnet, Corney, Kenney, and G. Kenney. Sister Hutty is matron of the local V.A.D. Hospital.

The above named are the only ones that have been sent by the hospital committee, and are entitled to return to their posts after the war. Many others left at the conclusion of their training to do naval or military nursing.

